# MicroRNA expression profiling for the prediction of resistance to neoadjuvant radiochemotherapy in squamous cell carcinoma of the esophagus

**DOI:** 10.1186/s12967-018-1492-9

**Published:** 2018-04-25

**Authors:** Julia Slotta-Huspenina, Enken Drecoll, Marcus Feith, Daniel Habermehl, Stephanie Combs, Wilko Weichert, Marcus Bettstetter, Karen Becker, Rupert Langer

**Affiliations:** 1 Institute of Pathology, Technische Universität München, Trogerstrasse 18, 81675 Munich, Germany; 2 Department of Surgery, Klinikum Rechts der Isar, Technische Universität München, Ismaningerstrasse 22, 81675 Munich, Germany; 3 Department of Radiation Oncology, Klinikum Rechts der Isar, Technische Universität München, Ismaningerstrasse 22, 81675 Munich, Germany; 4 Teilgemeinschaftspraxis Molekularpathologie Südbayern, Giesinger Bahnhofplatz 2, 81539 Munich, Germany; 5 Institute of Pathology, University of Bern, Murtenstrasse 31, 3008 Bern, Switzerland

**Keywords:** Esophagus, Squamous cell carcinoma, miRNA, Neoadjuvant therapy, Response

## Abstract

**Background:**

MicroRNAs (miRNAs) play an important role in cancer biology. Neoadjuvant radiochemotherapy followed by surgery is a standard treatment for locally advanced esophageal squamous cell carcinoma (ESCC). However, a subset of patients do not respond. We evaluated whether miRNA profiles can predict resistance to radiochemotherapy.

**Methods:**

Formalin-fixed, paraffin-embedded pretherapeutic biopsies of patients treated by radiochemotherapy followed by esophagectomy were analyzed. The response was determined by histopathological tumor regression grading. miRNA profiling was performed by microarray analysis (Agilent platform) in 16 non-responders and 15 responders. Differentially expressed miRNAs were confirmed by real-time quantitative PCR (qRT-PCR) in an expanded cohort of 53 cases.

**Results:**

The miRNA profiles within and between non-responders and responders were highly similar (r = 0.96, 0.94 and 0.95). However, 12 miRNAs were differentially expressed (> twofold; p ≤ 0.025): non-responders showed upregulation of hsa-miR-1323, hsa-miR-3678-3p, hsv2-miR-H7-3p, hsa-miR-194*, hsa-miR-3152, kshv-miR-K12-4-3p, hsa-miR-665 and hsa-miR-3659 and downregulation of hsa-miR-126*, hsa-miR-484, hsa-miR-330-3p and hsa-miR-3653. qRT-PCR analysis confirmed the microarray findings for hsa-miR-194* and hsa-miR-665 (p < 0.001 each) with AUC values of 0.811 (95% CI 0.694–0.927) and 0.817 (95% CI 0.704–0.930), respectively, in ROC analysis.

**Conclusions:**

Our results indicate that miRNAs are involved in the therapeutic response in ESCC and suggest that miRNA profiles could facilitate pretherapeutic patient selection.

**Electronic supplementary material:**

The online version of this article (10.1186/s12967-018-1492-9) contains supplementary material, which is available to authorized users.

## Background

Esophageal squamous cell carcinoma (ESCC) is one of the most aggressive tumor types with an extremely high lethality and low survival rates. Despite the increasing incidence of esophageal adenocarcinoma (EAC), especially in Western countries, ESCC remains the predominant tumor type worldwide and has a substantial prevalence in Europe and North America [1, 2]. Multimodal treatment, usually consisting of neoadjuvant radiochemotherapy (RCT) followed by surgery, leads to a survival benefit compared with surgery alone for locally advanced ESCC [3–7]. However, many patients do not show a measurable tumor response to neoadjuvant therapy, and the prognosis of these patients in the combined setting may be even worse than with surgery alone due to the side effects of cytotoxic treatment and delay of intervention [8–10]. Conversely, definitive radio- or radiochemotherapy represents an attractive alternative option for patients who are not suitable for major surgical therapy due to their medical status [11]. Therefore, pretherapeutic identification of patients who would not respond to neoadjuvant or definitive radiotherapy is of major interest for clinical decision-making and individualizing therapy.

Analysis of microRNAs (miRNAs) has strong potential for the identification of novel prognostic or predictive biomarkers. miRNAs are single-stranded, non-coding, highly conserved RNA molecules with a length of 20–25 nucleotides [12, 13]. miRNAs regulate the expression of genes via highly specific binding on messenger RNA (mRNA). Since the first description of miRNAs approximately 20 years ago, more than 17,000 miRNAs have been detected in various species, and 1900 of them are observed in the human genome. Importantly, more than half of all genes of the genome are suspected to be regulated by miRNAs, indicating the importance of miRNAs for the control of growth, differentiation and function of cells [12, 14, 15]. In addition to neurodegenerative diseases, infections and immune-related diseases, cancer has been the most prominent of human diseases with a clear role for miRNA regulation since the beginning of miRNA research. On this subject, miRNAs are involved in carcinogenesis, cancer progression, and therapy resistance and response [16, 17].

Although several studies have investigated the impact of miRNA expression on the response to cytotoxic treatment in esophageal cancer in vitro and ex vivo in patient tissue collections (for review see [18]), to date, no constant results or reproducible tissue-generated data regarding differentially expressed miRNAs have been published.

We present a microarray-based approach to identify miRNA profiles that differ between responders and non-responders in pretherapeutic biopsies from patients with ESCC after neoadjuvant RCT and surgery. Microarray data were confirmed by quantitative RT-PCR for differentially expressed miRNAs in an expanded cohort.

## Methods

### Patient characteristics and tissue specimens

FFPE tumor samples from 53 patients with locally advanced esophageal squamous cell carcinoma were investigated. The patients were treated between 1996 and 2009 at the Department of Radiation Oncology and Department of Surgery at the Klinikum Rechts der Isar der Technischen Universität München, Germany. The median age of the patients was 57.3 (range: 29–71 years) with 11 female (20.8%) and 42 male patients (79.2%), reflecting the expected gender distribution for this tumor. Tumor differentiation was G2 (moderately differentiated) in 20 cases (37.7%) and G3 (poorly differentiated) in 33 cases (62.3%).

Preoperative RCT consisted of simultaneously applied cisplatin (15 patients) or oxaliplatin (38 patients), and 5-fluorouracil based chemotherapy and external-beam radiotherapy radiation with overall doses of 30–60 Gy, predominantly 45 Gy (45 patients; single doses of 1.8–2 Gy) [8, 19]. Surgery was performed 4–6 weeks thereafter with esophagectomy as described in detail previously [8, 20]. Tumor regression grade (TRG) was histologically assessed in posttreatment resection specimens in a standardized way by microscopic evaluation of the complete previous tumor bed, as described previously [8, 21]. Cases with no residual tumor were classified as “responders” (TRG 1a) and cases with > 50% residual tumors were classified as “non-responders” (TRG 3). Tumors with incomplete or partial regression (1–50% residual tumors) were not included in this study because the data about the prognostic impact of incomplete and partial regression in the literature do not show consistent results [8, 22]. Postoperative histopathological findings after neoadjuvant radiochemotherapy are given in Table 1. Overall survival (OS) was calculated from the day of surgery to death.

**Table 1:** Postoperative findings after neoadjuvant radiochemotherapy

Factor	n	%
Tumor regression grade		
TRG 1a	26	49
TRG 3	27	51
UICC ypT category		
ypT0	25	47
ypT1	2	4
ypT2	3	6
ypT3	18	34
ypT4	5	9
Lymph node status		
ypN0	32	60
ypN1	21	40
Distant metastasis		
M0	51	96
M1	2	4
Resection status		
R0	39	74
R1	14	26

Preoperative biopsies from 31 patients containing sufficient tumor material was used for microarray analysis. Confirmation by quantitative RT-PCR (qRT-PCR) was performed on an extended cohort including the cases from the microarray analysis and biopsy tissue from another 22 patients.

### RNA extraction

The tumor cell content of a minimum of 80% of the biopsies was required, and manual microdissection was performed to enrich the tumor content when necessary. Total RNA, including small RNAs, was extracted using the FFPE miRNeasy Kit (Qiagen, Hilden, Germany). For each sample, between 2 and 10 unstained 10-µm sections were manually microdissected after deparaffinization, and RNA was extracted using 150 µl of proteinase K digestion buffer according to the manufacturer’s instructions. Total RNA was measured using the NanoDrop photospectrometer (NanoDrop, Wilmington, DE, USA) and was further processed if the A260/A280 ratio was ≥ 1.8 and the A260/A230 ratio was ≥ 1.4. All samples were analyzed using RNA 6000 Nano LabChip Kits (Agilent Technologies).

### Preparation of cyanine-3 labeled miRNA and microarray hybridization

The total RNA samples were spiked with in vitro-synthesized oligonucleotides (MicroRNA Spike-In Kit, Agilent Technologies). The spiked total RNA was treated with alkaline calf intestine phosphatase (CIP). Subsequently, the dephosphorylated RNA was labeled (miRNA Complete Labeling and Hyb Kit, Agilent Technologies) using the T4 RNA ligase, incorporating Cyanine-3-pCp. Next, 100 ng of total RNA per sample were introduced into the labeling reaction. After clean-up, Cyanine-3-labeled miRNA samples were prepared for one-color-based hybridization (Complete miRNA Labeling and Hyb Kit; Agilent Technologies). Each Cyanine-3-labeled miRNA sample was hybridized for 20 h at 55 °C on separate Human miRNA Microarrays, Release 16.0 (Agilent Technologies; AMADID 031181, 8 × 60 K format), containing probes for 1205 human and 144 human viral miRNAs. Thereafter, the microarrays were washed with increasing stringency using Gene Expression Wash Buffers (Agilent Technologies) followed by drying with acetonitrile (SIGMA). Fluorescent signal intensities were detected using Scan Control A.8.4.1 Software (Agilent Technologies) in the Agilent DNA Microarray Scanner.

### Quantitative RT-PCR

For validation of the microarray data, differentially expressed miRNAs were investigated by qRT-PCR using the miRCURY LNA™ Universal RT microRNA PCR system (Exiqon A/S, Denmark). Details regarding the commercially available miRNA primer sets used and sequences for individual Custom PCR primer sets are given as Additional file 1. The cDNA samples were prepared using 20 ng of total RNA and the Universal cDNA synthesis kit (Exiqon A/S, Vedbaek, Denmark) according to the manufacturer’s recommendations. A 10-µl volume of a 50× dilution of cDNA was used in each of the real-time PCR reactions with SYBR^®^ green master mix and miRNA LNA™ PCR primer sets (both from Exiqon A/S) following the manufacturer’s instructions. All samples were run in triplicate. Real-time PCR was carried out on a Light Cycler 480 instrument (Roche Diagnostics), and the arithmetic mean of each triplicate measurement was used for further analysis. The relative quantification of miRNA expression was performed using a non-linear algorithm (second derivative maximum, Roche LightCycler Software V1.5) with a standard curve for each individual assay to calculate and correct for the amplification efficiency and using SNORD 44 as a reference for normalization.

To minimize the data variation in separate runs, a pool of 10 non-tumor samples was examined on the same runs. A no-reverse-transcriptase control (no RT) was included for each run of real-time RT-PCR to ensure that the RNA samples were not contaminated with genomic DNA.

### Statistical analysis

For the microarray analysis, the software tools Feature Extraction 10.7.3.1 and GeneSpring GX 11.5 were used for quality control, statistical data analysis, miRNA annotation and visualization. A detailed description of the statistical workup of the microarray data is given as Additional file 2.

For statistical analysis of the confirmation experiments, SPSS software (IBM SPSS statistics version 24 was used. The associations between groups of patients were given in cross tabs, and differences were determined using χ^2^-test. Comparisons between groups were performed using the non-parametric Mann–Whitney U test and results were adjusted for multiple testing using the Bonferroni correction, where appropriate. Categorization into high and low miRNA-expressing tumors was conducted according the results of ROC analysis (non-response as reference). For survival analysis, the log-rank tests and Kaplan–Meier curves were used, as well as Cox proportional hazard tests for multivariate analysis. All tests were two-sided, and the significance level was set to p < 0.05 or lower, in line with the correction for multiple testing.

## Results

### Microarray analysis

**Fig. 1:**
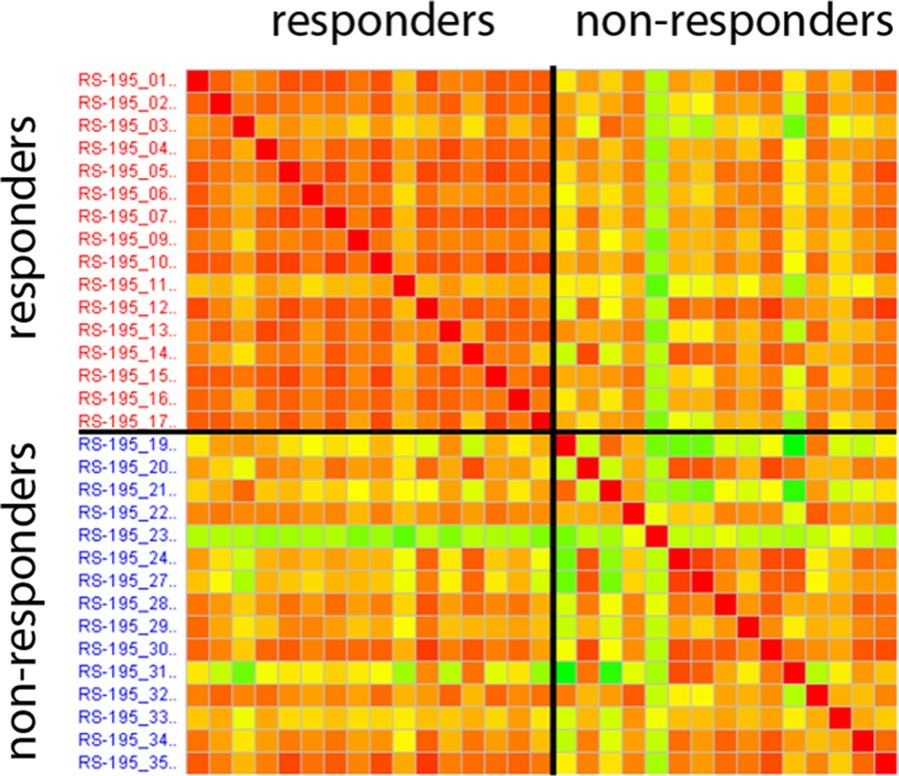
Correlation of miRNA expression. Heat-map plot for the correlation coefficients r of the miRNA expression profiles among all samples analyzed by microarray analysis [red color r = 1.00 (best), green color r = 0.85 (worst)]

Thirty-one biopsy samples (16 responders and 15 non-responders) passed the quality check and could be used for microarray analysis. In general, the miRNA expression profiles in the ESCC biopsies examined were quite similar, both within the group of responders and non-responders: Correlation coefficients (r) within the group of responders ranged from 0.93 to 0.98 (average r = 0.96) and within the group of non-responders from 0.85 to 0.98 (average r = 0.94). Moreover, the miRNA profiles were rather similar between responders and non-responders with correlation coefficients ranging from 0.88 to 0.98 (average r = 0.95). The correlation (r) of the samples is visualized in a heat-map (Fig. 1).

Despite the quite homogeneous miRNA expression profile in pretherapeutic biopsies, a small number miRNAs fulfilled the strict predefined criteria for being designated as differentially expressed between responders and non-responders. The following eight miRNAs were upregulated in non-responders: hsa-miR-1323, hsa-miR-3678-3p, hsv2-miR-H7-3p, hsa-miR-194*, hsa-miR-3152, kshv-miR-K12-4-3p, hsa-miR-665 and hsa-miR-3659. By contrast, the four miRNAs hsa-miR-126*, hsa-miR-484, hsa-miR-330-3p and hsa-miR-3653 were downregulated in non-responders compared with that in responders (Table 2).

**Table 2:** Differentially expressed miRNAs as identified by microarray analysis in non-responders compared with that in responders

miRNA	Regulation in non-responders	Fold chance	p value
miR-3678-3p	Upregulated	10.774	0.022
miR-3152	Upregulated	5.732	0.018
kshv-miR-K12-4-3p	Upregulated	3.211	0.006
miR-1323	Upregulated	22.143	0.004
miR-665	Upregulated	2.527	0.004
hsv2-miR-H7-3p	Upregulated	6.864	0.002
miR-194*	Upregulated	5.820	0.001
miR-3659	Upregulated	2.227	0.000
miR-126*	Downregulated	− 10.462	0.006
miR-484	Downregulated	− 6.956	0.021
miR-330-3p	Downregulated	− 6.609	0.029
miR-3653	Downregulated	− 1.909	0.022

The complete list of analyzed miRNAs, their expression levels and respective p values for the comparison of the expression levels between responders and non-responders are given in Additional file 3.

### Confirmation by single real-time RT PCR

**Fig. 2:**
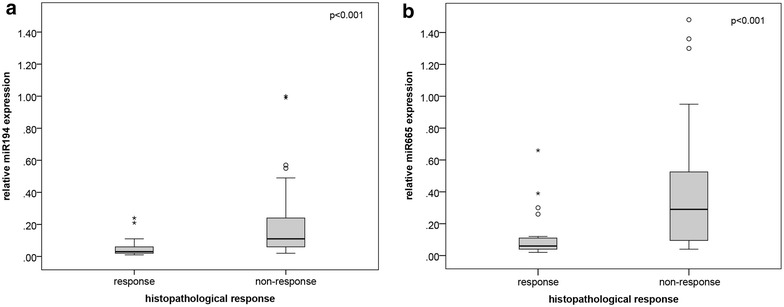
Expression levels of miRNA-194 and miRNA-665 in ESCC samples. Box plots illustrating miRNA expression levels quantified by single qRT-PCR for miR-194* (**a**) and miR-665 (**b**) in pretherapeutic biopsies of 26 responders and 27 non-responders

Real-time quantitative PCR for the confirmation of microarray analysis findings was performed for 12 differentially expressed miRNAs in the expanded collective of 53 biopsy samples. However, four miRNAs (hsa-miR-1323, hsa-miR-3678-3p, hsa-miR-3152 and kshv-miR-k12-4-3p) could be detected at only very low levels by qRT-PCR, making valid quantification impossible, and the miRNAs hsa-miR-126*, hsa-miR-484, hsa-miR-330-3p, hsa-miR-3653, hsa-miR-194*, hsv2-miR-H7-3p, hsa-miR-665 and hsa-miR-3659 could be constantly detected in all samples and quantified. For miR-194* and miR-665 but not the remaining miRNAs, the microarray results could be confirmed with significantly higher miRNA levels in non-responders than in responders (median quantitative gene expression levels: 0.11 vs. 0.03, respectively, and 0.29 vs. 0.06, respectively; p < 0.001 each with a significance level of p < 0.006 after correction for multiple testing, Fig. 2). ROC analysis showed AUC values of 0.811 (95% CI 0.694–0.927) for miR-194* and 0.817 (95% CI 0.704–0.930) for miR-665 for the non-response to RCTX. The combination of miR-194* and miR-665 (sum of relative values in relation to the median expression level) had only a slightly better AUC value of 0.824 (95% CI 0.713–0.935). Crosstab analysis using the cut-offs defined by ROC analysis confirmed the association between high levels of miR-194* and miR-665 and non-response to neoadjuvant RCTX (p < 0.001 and p = 0.006; Table 3). Interestingly, for the miRNAs that were downregulated in non-responders, microarray results could not be confirmed in single qRT-PCR analysis.

**Table 3:** Association between the miRNA expression levels and response to radiochemotherapy in pretherapeutic ESCC biopsy samples

	Response (TRG1a)	Non-response (TRG3)	Total	p value
High miRNA-194*	21	7	28	< 0.001
Low miRNA-194*	5	20	25	
Total	26	27	53	
High miRNA-665	19	9	28	0.006
Low miRNA-665	5	20	25	
Total	26	29	53	

### Survival analysis

**Fig. 3:**
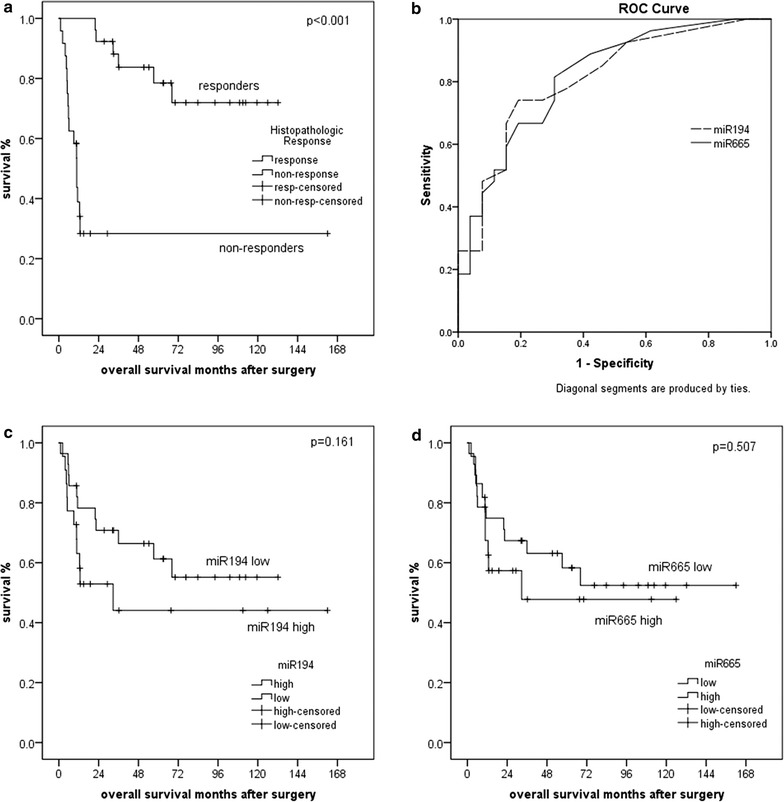
Survival and ROC analysis. Overall survival (OS) for 53 patients with respect to histopathologic tumor regression (**a**), miR-194* (**c**) and miR-665 expression (**d**). Receiver operating characteristic (ROC) curves (**b**) for the histopathological non-response to RCTX stratified by high miRNA-194* and miRNA-665 expression, respectively

Responders showed significantly better overall survival (OS) than non-responders (p < 0.001), and responders significantly more often had lower ypT categories and the absence of lymph node or distant metastases than non-responders (p < 0.001 each). Higher levels of miR-194* and miR-665 defined by ROC analysis were associated only in trend with a worse outcome of the patients in univariate analysis (p = 0.052 and p = 0.197, respectively; Fig. 3). In multivariate analysis encompassing the relevant prognostic pathological parameters and miRNAs, only the ypN category emerged as constant independent prognostic factors (HR: 7.561; 95% CI 1.6–35.1; p = 0.010; Table 4).

**Table 4:** Multivariate Cox regression analysis (overall survival) for the histopathological response and non-response (tumor regression), pathologic tumor stage (UICC ypT category), lymph node status (ypN), distant metastasis, and miRNA-194* and miRNA-665 expression levels

Factor	HR	95.0% CI for HR		p value	p value
		Min	Max		Univariate
Tumor regression	5.681	0.507	63.709	0.159	< 0.001
UICC ypT category	1.460	0.724	2.947	0.290	< 0.001
Lymph node metastases	7.561	1.631	35.062	0.010	< 0.001
Distant metastasis	1.666	0.243	11.442	0.603	< 0.001
Resection status	0.384	0.112	1.315	0.128	0.0120
miRNA-194*	0.669	0.133	3.352	0.625	0.052
miRNA-665	0.634	0.104	3.861	0.621	0.197

### Comparison with previous miRNA studies of the response to neoadjuvant treatment

Ex vivo and in vitro studies with expression analyses of single miRNAs and miRNA microarray analyses have already been used by others to identify miRNA profiles in esophageal carcinomas that are potentially predictive for the response or resistance to chemo- or radiochemotherapy [23–32]. There is considerable variety in the platforms used, tissue analyzed (i.e., FFPE vs. fresh (unfixed) tissue), case collection or neoadjuvant treatment. We screened the results of our microarray analysis to compare the results of these studies with the present investigation. An overview of miRNAs that have been described as differentially expressed between responding and non-responding esophageal carcinomas in relation to the results of the present work is given in Additional file 4.

## Discussion

In this tissue-based study, we could demonstrate the feasibility to apply microarray-based miRNA profiling on the FFPE tissue of ESCC prior to neoadjuvant RCT. Although the tumors harbored very homogeneous miRNA expression profiles in general, we could identify several miRNAs that were differentially expressed in responders and non-responders.

One major advantage in the context of tissue-based expression analysis of miRNA is the stability of the small RNA fragments that undergo degradation by prior fixation (e.g., by formalin) to a considerably lesser degree than longer mRNA sequences. The availability of unfixed fresh tumor material for molecular analysis is often limited. Therefore, the use of FFPE tissue, which is routinely used for the histopathological diagnostic process, would offer major advantages for retrospective and prospective molecular studies and, in future perspectives, for the application within a diagnostic process. The comparability between fixed and unfixed tissue for miRNA analysis has been shown in several studies [33–38]. At least, to the best of our knowledge, our study on 31 samples is currently the largest application of FFPE tissue in bioptic esophageal cancer tissue prior to preoperative treatment to identify differentially expressed miRNAs.

By microarray analysis, 12 miRNAs were identified to be different between responders and non-responders. Eight miRNAs were up regulated, and four miRNAs were down regulated, in non-responders. We could confirm the microarray results by single real-time RT-PCR for two miRNAs (miR-194* and miR-665) in an expanded case collection. Interestingly, based on a Pubmed literature research, we could not find any of the two confirmed miRNAs being described in association with a treatment response in tissue based studies before, neither in esophageal cancer nor in other tumor types. In our study, ROC analysis identified significant predictive cut-offs for a response. This underlines the potential impact of these miRNAs for the resistance of ESCC to radiochemotherapy.

miRNAs have already been investigated by others regarding similar research questions. Interestingly, many of the miRNAs that have been described to play a potential prognostic or predictive role in esophageal cancer in these studies (both adeno- and squamous cell carcinomas) could be detected by our microarray analysis. However, none of them showed the same differential expression between responders and non-responders. This may be due to diverging case collections, with mixed adeno- and squamous cell carcinomas [39, 40] or purely adenocarcinomas, or due to the variation in neoadjuvant therapy protocols (radiochemotherapy chemotherapy only), or due to the different ethnic backgrounds of the patient population (Western and Asian populations) [23]. Of note, one recent paper that also used the Agilent detection platform showed that a model encompassing upregulated miR-145-5p, upregulated miR-152, downregulated miR-193b-3p, and upregulated miR-376a-3p was highly accurate for the prediction of a nonresponse to neoadjuvant radiochemotherapy in an Asian cohort [23]. We could not confirm these findings in our analysis, and conversely, the most significantly deregulated miRNAs of our study did not show up in this paper. Another aspect that could explain these divergent results may be a technical issue: different platforms for comprehensive miRNA expression analysis may provide different results [41, 42].

Despite these discrepancies, however, the results of these and our studies again highlight a potential promising role of miRNA profiling but also point to the need of standardization of biomarker research before potential application in clinical practice.

Our study may be limited by the relatively small sample size and lack of a true external validation cohort. The small case numbers may also explain that we did not find a correlation between the miRNAs and patient outcome. However, we present a homogeneous case collection with standardized assessment of tumor regression as a surrogate for the treatment response, and we concentrated on patients with complete regression versus clear non-responders with > 50% residual tumors. Data from literature are not consistent with regard to the prognostic value of partial tumor regression [8, 22] and we wanted to examine two extreme prognostic groups in view of the limited sample size. We deliberately accepted a potential bias caused by this restrictive approach that also may influence survival results due the patient’s selection prior to the tissue analysis. Our primary goal, though, was to perform a pilot study for the technical application of microarray miRNA profiling on clinical FFPE tissue samples. The results of this part could be at least partially confirmed using a different detection method in an expanded case collection. However, a validation of our results using a larger case collection is clearly demanded. A second limitation may be the lack of a comprehensive mutational analysis of the tumors. miRNAs closely interact with both wild-type and mutated genes. Further analysis should also integrate the interaction and networks between therapy-related genes and miRNAs, a phenomenon that could outrange the impact of a solely miRNA-based study alone.

## Conclusions

Taken together, our data demonstrate the feasibility of microarray-based miRNA analysis of diagnostic FFPE bioptic tumor tissue. Although several studies have suggested that miRNAs might be involved in the modulation of the therapy response and that miRNAs could be used as a predictive biomarker, there are major discrepancies among the studies published to date. Therefore, validation of our data and those from others is essential before implementing miRNA expression analysis as a tool for molecular response prediction in ESSC patients. This finding may be of interest, because most recently, the determination of circulating (tumoral) miRNAs in blood samples has been shown to be a feasible approach as a potential tool in cancer diagnostics [43–45]. Prior identification of tissue-derived data may serve as the basis for the further development of such non-invasive tests for response prediction and monitoring of therapy.

## Additional files

**Additional file 1.** Target sequences of miRNAs.

**Additional file 2.** Statistical approach of microarray analysis.

**Additional file 3.** Complete results of microarray analysis.

**Additional file 4.** Comparison of published miRNA/miRNA array data with the results of the microarray analysis of the present study.
