# Correction to: MicroRNA expression profiling for the prediction of resistance to neoadjuvant radiochemotherapy in squamous cell carcinoma of the esophagus

**DOI:** 10.1186/s12967-018-1513-8

**Published:** 2018-05-16

**Authors:** Julia Slotta-Huspenina, Enken Drecoll, Marcus Feith, Daniel Habermehl, Stephanie E. Combs, Wilko Weichert, Marcus Bettstetter, Karen Becker, Rupert Langer

**Affiliations:** 1 Institute of Pathology, Technische Universität München, Trogerstrasse 18, 81675 Munich, Germany; 2 Department of Surgery, Klinikum Rechts der Isar, Technische Universität München, Ismaningerstrasse 22, 81675 Munich, Germany; 3 Department of Radiation Oncology, Klinikum Rechts der Isar, Technische Universität München, Ismaningerstrasse 22, 81675 Munich, Germany; 4 Department of Radiation Sciences (DRS), Institute of Innovative Radiotherapie (iRT), Helmholtz Zentrum München, Ingolstädter Landstrasse 1, 85764 Neuherberg, Germany; 5 Deutsches Konsortium für Translationale Krebsforschung (DKTK), Partner Site Munich, Munich, Germany; 6 Teilgemeinschaftspraxis Molekularpathologie Südbayern, Giesinger Bahnhofplatz 2, 81539 Munich, Germany; 7 Institute of Pathology, University of Bern, Murtenstrasse 31, 3008 Bern, Switzerland

## Correction to: J Transl Med (2018) 16:109 10.1186/s12967-018-1492-9

Following publication of the original article [1], the authors reported that for one of the authors, Stephanie E. Combs, the middle name was accidentally omitted. They also reported that for two of the authors, Daniel Habermehl and Stephanie E. Combs, two affiliations were accidentally omitted. In this Correction the incorrect and correct author name are shown and the two omitted affiliations are listed.

Originally the author name has been published as:

- Stephanie Combs

The correct author name is:

- Stephanie E. Combs

The two missing affiliations for Daniel Habermehl and Stephanie E. Combs are:

- Department of Radiation Sciences (DRS), Institute of Innovative Radiotherapie (iRT), Helmholtz Zentrum München, Ingolstädter Landstrasse 1, 85764 Neuherberg, Germany.
- Deutsches Konsortium für Translationale Krebsforschung (DKTK), Partner Site Munich, Munich Germany.

The complete list of affiliations is included in this Correction.
