# Supplementary material for: MicroRNA expression profiling for the prediction of resistance to neoadjuvant radiochemotherapy in squamous cell carcinoma of the esophagus
Source: J Transl Med. 2018 Apr 25;16:109. doi: 10.1186/s12967-018-1492-9 (PMC5918871; doi:10.1186/s12967-018-1492-9)
Supplement: Supplementary file 1 — Additional file 1. Target sequences of miRNAs. [file 12967_2018_1492_MOESM1_ESM.docx]

**Supplemental file 1: target sequences of miRNAs**

| **miRNA** | **target sequence** | **product number (Exiqon)** |
| --- | --- | --- |
| miR-1323 | UCAAAACUGAGGGGCAUUUUCU |  |
| miR-3678-3p | CUGCAGAGUUUGUACGGACCGG |  |
| hsv2-miR-H7-3p | UUUGGAUUCCGACCCCUCGUC |  |
| miR-194* | CCAGUGGGGCUGCUGUUAUCUG | 202873 |
| miR-3152 | UGUGUUAGAAUAGGGGCAAUAA |  |
| kshv-miR-k12-4-3p | UAGAAUACUGAGGCCUAGCUGA |  |
| miR-665 | ACCAGGAGGCUGAGGCCCCU | 202587 |
| miR-3659 | UGAGUGUUGUCUACGAGGGCA |  |
| miR-126* | UCGUACCGUGAGUAAUAAUGCG | 204584 |
| miR-484 | UCAGGCUCAGUCCCCUCCCGAU | 202351 |
| miR-330-3p | GCAAAGCACACGGCCUGCAGAGA | 202075 |
| miR-3653 | CUAAGAAGUUGACUGAAG |  |
| SNORD44 (hsa) |  | 203902 |
