# Supplementary material for: MicroRNA expression profiling for the prediction of resistance to neoadjuvant radiochemotherapy in squamous cell carcinoma of the esophagus
Source: J Transl Med. 2018 Apr 25;16:109. doi: 10.1186/s12967-018-1492-9 (PMC5918871; doi:10.1186/s12967-018-1492-9)
Supplement: Supplementary file 2 — Additional file 2. Statistical approach of microarray analysis. [file 12967_2018_1492_MOESM2_ESM.docx]

**Supplemental File 2**

***Microarray Analysis - Statistics***

For the microarray analysis the software tools Feature Extraction 10.7.3.1 and GeneSpring GX 11.5 were used for quality control, statistical data analysis, miRNA annotation and visualization. A detailed description of the statistical analysis of the microarray data is given as supplemental data. Quantile normalization was applied to the data set imposing the same distribution of probe signal intensities for each array. After normalization, data were returned as log2 transformed values and filtered based on flags. The values were then visualized as raw data (before normalization) or in a log2 transformed manner (after normalization).

The similarity between different samples based on global gene expression profiles was assessed by correlation analysis. Pearson’s correlation coefficients were calculated for all biological replicates within the groups and for all pair-wise comparisons of the samples in the experiment. The quantile normalized, log2 transformed data were averaged across replicates. Welch’s approximate t-test (“unpaired unequal variance”, parametric) was applied to the comparison of the different groups. In addition, The Welch’s approximate t-test-based p-value was corrected for multiple testing using the algorithm of Benjamini and Hochberg (Hochberg and Benjamini. More powerful procedures for multiple significance testing. Stat Med. 1990 Jul;9(7):811-8).

Responders and nonresponders were compared in a pairwise manner. The extent and direction of differential expression between the groups was determined by calculating a fold change value. First, the averaged normalized signal values were transformed from the log2 to the linear scale. Then, their ratio was calculated. This way of calculation has the effect that the up-regulated miRNAs have ratios ranging from > 1 to + ∞, which are identical to the Fold Change value. For the down-regulated miRNAs, the range of ratios is compressed between 0 and < 1. To achieve Fold Change values with an equal order of magnitude for both, up- and down-regulation, the ratios ranging from 0 to < 1 were then transformed to the linear scale.

In order to filter for miRNAs with a robust differential expression between two compared groups, a probe was only considered if it is reliably detectable in all samples of one of the two compared groups. For assuring statistical significancy, a stringent filtering approach was used, which determined a miRNA only classified as differentially expressed if its corrected p-value is ≤ 0.025, if it has a fold change value ≥ 2.0 and it was detected in all samples of the compared groups.
